# Wicked problems: a value chain approach from Vietnam’s dairy product

**DOI:** 10.1186/2193-1801-2-161

**Published:** 2013-04-15

**Authors:** Nguyen Viet Khoi

**Affiliations:** VNU University of Economics and Business, 144 Xuan Thuy, Cau Giay, Hanoi, Vietnam; Columbia Business School, Columbia University, New York, USA

**Keywords:** Value chain analysis, Dairy industry, Vietnam’s dairy industry, Production management

## Abstract

M10, M11

**Electronic supplementary material:**

The online version of this article (doi:10.1186/2193-1801-2-161) contains supplementary material, which is available to authorized users.

## Mapping the value chain of Vietnam’s dairy industry

**Figure 1:**
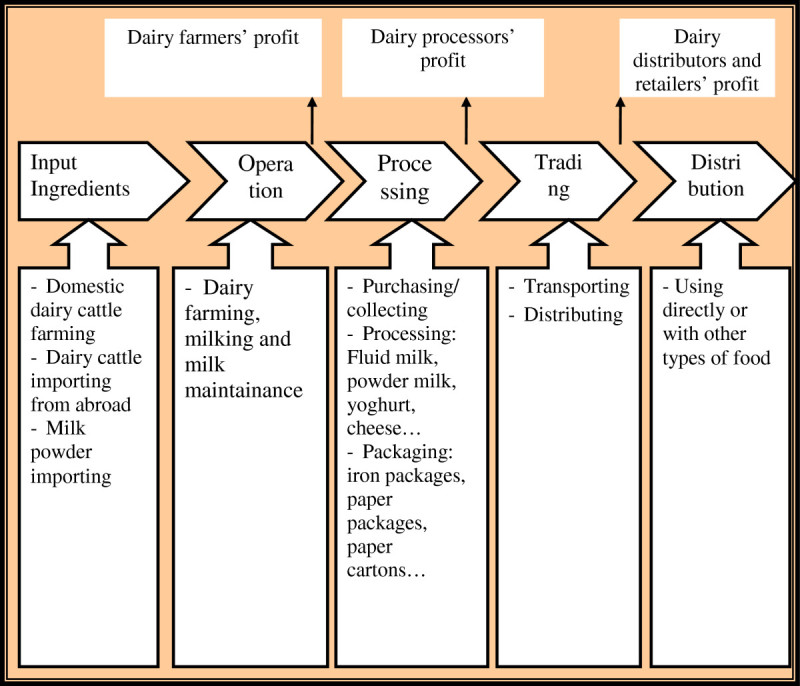
**The value chain map of Vietnam’s dairy industry.**

Each industry has their own value chain systems and these value chains will also be adjusted due to regional characteristics. The value chain of Vietnam’s dairy sector consists of five activities as: ingredients, production, processing, marketing and consumption (See Figure1 The value chain map of Vietnam’s dairy industry). We will be able to understand these activities by analyzing each activity and player participating in these value chains. By thoroughly examine the two most important activities, milk production and processing, we will have a clear answer why there still existed inadequacies of value added creation in these two activities. In addition, this research does not carry out on humans, which does not have to be in compliance with the Helsinki Declaration. And this research does not contain any experimental research on animals, which does not need to follow internationally recognized guidelines.

### Activity 1: Input ingredients include the import of dairy breeds for husbandry and the purchasing of raw milk powder ingredients for processing activities

**Figure 2:**
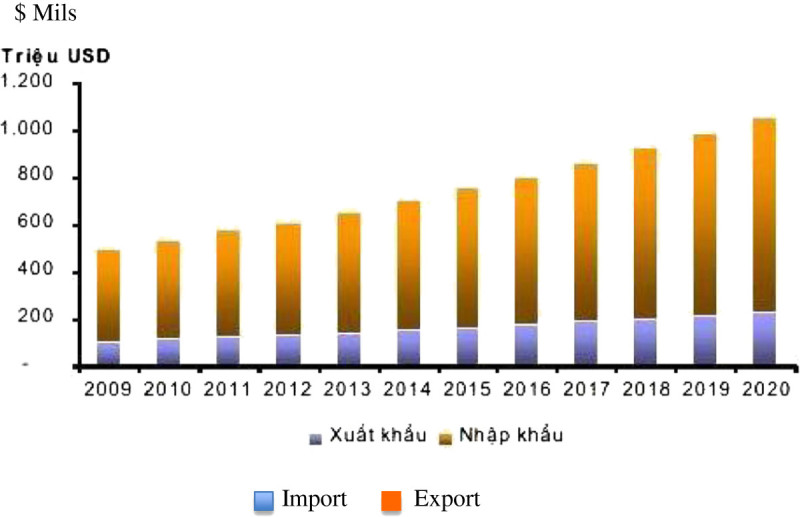
**The import and export turnover of dairy cattle.**

In dairy farming, the import turnover of dairy cattle is currently 3.5 times as much as the export turnover. (*See the chart of import and export turnover of dairy cattle since 2009 and forecast until 2020 - Figure*2).

Since the dairy farming is underdeveloped, Vietnam has to import a large quantity of milk powder materials to serve processing activities. It is estimated that the imported milk powder materials account for 70% of the demand for milk powder. Thus, the import of dairy cattle and milk powder materials shows significant dependence of Vietnam on the world’s material resources, which affects the sustainable development of the dairy industry. Therefore, in input ingredients, the dairy sector has been seeking permanent solutions like improving dairy cattle breed selection and crossing to increase milk quantity and productivity.

### Activity 2: Production activity includes dairy cattle husbandry, milking and milk maintenance

This activity is the worst one of the value chain as it is now facing the following difficulties:

Large initial investment: Estimatedly, a dairy cow has a relatively high price of approximately 20-30 million VND/head. In addition, dairy cattle are in need of special care. Apart from basic conditions such as spacy and prevailing holding pens, well-equipped cooling systems and milking machines, the feed requirements are imposed very strict. These factors entail a large amount of capital invested from dairy farmers. However, in the current animal husbandry system of Vietnam, there are up to 95% of small-scale dairy farming households (with only 8 - 10 cows). Therefore, farmers do not meet the capital requirements to ensure adequate facilities for their dairy farming.

Furthermore, a lot of difficulties can be seen regarding the supply of feed. The amount of natural grass and grown grass only satisfies about 30% forage needs of dairy cattle. Additionally, the expansion of grasslands encounters great difficulties: in major cities and towns, the price of land is the biggest obstacle for farmers; meanwhile, the land-abundant areas are not qualified enough to develop dairy farming. Some cattle farming regions mainly depend on poor-quality pastures which are unsafe for the health of dairy cows due to the effect of chemicals used in herbicides, insecticides or other hazardous waste of industrial plants. Insufficient raw green feed causes farmers to increase the proportion of processed feed – which affects milk quality. In addition to that, the dairy industry “encounters” the shortage of specialized dairy farmers. Realizing that dairy farming brings economic efficiency, farmers rush into making investment decision without acquiring essential farming knowledge. Besides, local authorities do not provide adequate support, which leads to the low effectiveness of dairy cattle farming.

As a result of an inefficient dairy farming sector, the dairy industry now meets only 25% of the demand for fresh milk consumption in the market. The following table compares the production and consumption of fresh milk in recent years (See Table 1 The production and consumption of fresh milk and % satisfaction of domestic demand):

**Table 1:** **The production and consumption of fresh milk and % satisfaction of domestic demand**

Year	Milk production (thousand tons/year)	Milk consumption (thousand tons/year)	Rate of domestic milk production as compared to the demand (%)
2005	197,679	1004	19,67
2006	215,953	1056	20,45
2007	234,438	1239	18,77
2008	262,160	1257	20,07
2009	278,190	-	-
2010	306	1224	25

Source: Do Kim Tuyen (2010), The development of dairy farming in Vietnam 2001 - 2009 and forecast 2010-2020, Vietnam Livestock Production Department.

Therefore, although the capacity to meet demand has increased over the years, the market-demand satisfying level is still not high. This suggests that the dairy industry has a lot of potentials for further development.

### Activity 3: The next activity in the value chain is processing. In this part, processing plants will collect, then process and package milk

Currently, three major players are involved into collecting milk, including enterprises purchasing through their intermediary agents as well as co-operatives (co-operatives are established only in the places where many smallholder dairy farmers are concentrated and raw milk will be bulked at co-operatives before being transported to processors) and purchasing/collecting system through private collectors. The method of purchasing/collecting milk through private collectors is the most popular at present because up to 95% of dairy farmers follow the small and dispersive scale of production. Moreover, milk purchasing/collecting system has been developed incoherently and unpromptedly. Besides, low preservation technology reduces the quality of raw milk before it is sold to processors.

Meanwhile, although the dairy farmers offer a low milk price, it “escalates” sharply because it is sold through many intermediaries. Vietnam has 2 main companies involved in to purchasing/collecting and processing milk, namely Vinamilk and Dutchlady occupying 60% and 25% respectively of the total amount of milk produced nationwide (Van Cai D^2009^). This leads to monopoly pricing behaviors in collecting milk. In January 2011, the highest collecting price of processors was 11.500VND/kg. However, after milk was pasteurized, the price of milk increased by 2.5 times. The milk collecting method between Companies and dairy farmers is not transparent and fair, which makes the farmers feel they suffer from disadvantages. Although the input milk standard set by processors is not high, 20-50% of milk does not satisfy the quality requirement (according to the statistics of Vinamilk at the first half of 2008). Dairy farmers suspects about the milk evaluation of processors because the quality assessment procedure is conducted without their witness. This is “potential controversy” between sellers and buyers. Therefore, it is necessary to improve and enhance the mutual understanding between dairy farmers and processing plants to increase two sides’ profits.

Notably, the returning on investment of processors is quite high and assessed the highest in the whole value chain (See Table 2 The returning on investment of processing plants based on types of dairy products).

**Table 2:** **The returning on investment of processing plants based on types of dairy products**

	% cost	% retailing price
Condensed milk	17%	12%
Liquid milk	48%	28%
Yogurt	54%	30%
Low-priced whole milk	22%	15%
Average-priced whole milk	86%	40%

Source: Jaccar Vietnam.

**Figure 3:**
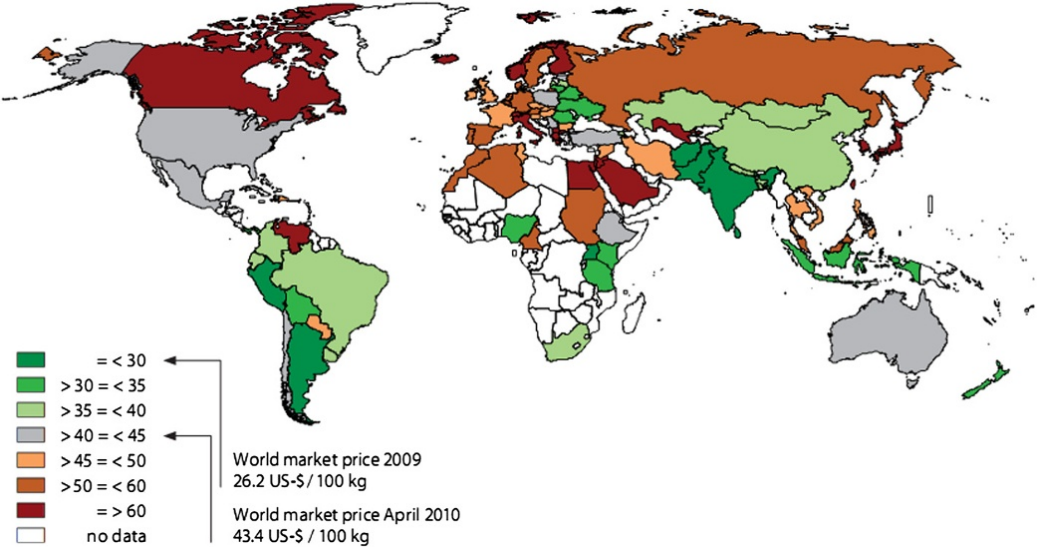
**World market price 2008 (USD).**

According to the authors, the average-priced powder milk generates the highest profit among dairy products due to some following reasons:

- At present, there are few mill processing plants. As a result, this easily leads to monopoly price set for milk products. Statistically, according to FAPRI Agricultural Outlook (2010), the Vietnamese dairy price level is high compared to the one of the regional countries (See Figure3 World market price 2008).

ECM stands for Energy Corrected Milk standardized at 4% fat and 3.3% protein.

- Psychologically, consumers tend to pay the higher price for dairy products containing nutritional components such as DHA, calcium and so on. Nevertheless, in fact, these nutrients only represent the small ratios of 1 kg milk. Meanwhile, their prices are not too high. For example, DHA, an expensive component of milk, occupies only the ratio from 0.2% to 0.5% of 1 kg milk. With the price around 80 USD/kg, the DHA per 1 kg milk costs around 5000-7000 VND (See Table 3 The price of some main components of milk). Therefore, the cost for nutritional components is remarkably small in comparison to the total one of milk product. The outstandingly higher milk price in Vietnam than in other regional countries helps to generate huge profits for dairy producers. According to the surveyed information of Jaccar investment fund, the return of dairy producers from average-priced powder milk achieves 86% compared to production cost.

**Table 3:** **The price of some main components of milk**

Component	Price (USD)/ton	Price (VND)/kg
The prices of milks with imported ingredients		
Full-cream milk powder	3.400 USD/ton	61.200
Skimmed milk powder	3.000 USD/ton	54.000
High-protein fat milk	5.000 USD/ton	90.000
The prices of some other components		
DHA	80 USD/kg	1.440.000
Calcium	7 USD/kg	126.000
Complex vitamin	10 USD/kg	180.000

Source: Trương Minh Huy (2009), Dairy sector value chain analysis, Research Department - Tai Viet Jointstock company, Hanoi, p.12.

In conclusion, after processing activity, dairy products prices are much higher than the collection one. As a result, this activity is considered to create the highest added value for dairy products.

### Activity 4: There are, currently, two major dairy distribution channels, namely traditional trade (distributors- wholesalers- retailers-consumers) and modern one (distributors-supermarkets-consumers)

According to the survey conducted by Jaccar investment fund, the ROI of distributors per retailing price occupies 13.4% of retailing price.

### Activity 5: Dairy consumption

**Figure 4:**
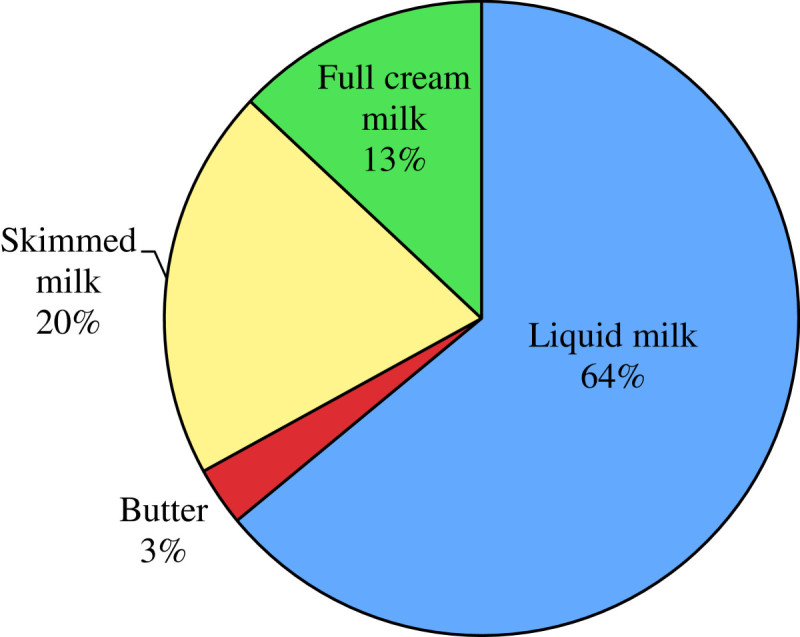
**The types of dairy products consumed in 2010.**

During recent years, dairy consumption of Vietnam market has been sharply increasing. 10% of Vietnamese population in the two big cities, namely Hochiminh City and Hanoi has been consuming 78% of dairy products. According to Habubank Securities' Dairy Sector Report, in (Habubank Securities^2009^), per capital average consumption of dairy products reached 14 liters/capita/year, which was fairly low in comparison to other country in the region (such as in Thailand 23 liters/capita/year or China 25 liters/capita/year). Nevertheless, this ratio has been considerably increased as compared to the ones of last years. Currently, the segment “liquid milk” still occupies the largest market share in Vietnamese dairy consumption demand thanks to its high nutritional value (See Figure4 The types of dairy products consumed in 2010). Moreover, it is easier to use this type of milk than others. However, due to high consumption demand and limited supply source, producers mainly imports powder milk from abroad and process it into fresh milk. This negatively affects the benefits of consumers.

In conclusion, dairy sector value chain analysis represents some remarkable points as follow:

- First, producing activity, namely cow farming is the weakest in the value chain at present.
- Second, the value of dairy products increases the most after processing activity.
- Third, the activities of the value chain have not been tightened with one another, especially between dairymen and processing plants. This causes some negative effects on the actors as well as the sustainable development of dairy sector.

## Measures

According to the aforementioned analysis, two packages of measures related to producing and processing activities have been recommended for the sustainable development of the dairy sector.

### The package of measures for milk producing

- Encouraging the support from local authorities: Currently, dairy cattle supplying source are mainly imported. Therefore, to help dairy cattle farming develop sustainably, local farmers should expand their breeding selection, breeding crossing. This will help to enhance the quality of breeding cow and reduce the dependence on external supplying source. Besides, training courses for dairy farming should be organized to enhance farmers’ knowledge about animal husbandry, which assists to improve the quality of raw milk.
- Local authorities should encourage local residents to raise dairy cattle in a larger scale. Centralized cow farming brings many benefits such as fully utilizing the economy of scale and reducing the cost per unit and so on. Therefore, it will help to increase milk productivity as well as profits from dairy cattle farming.
- Stable feed supplies will also bring enormous benefits for dairy cow farming. To the source of forage, it is essential to have effective solutions to develop grass system for dairy husbandry. Moreover, preserving feed source for dry season is necessary. To the source of corn/grain, authorities should intervene to stabilize the market and limit the monopoly pricing behaviors as well as regularly investigate in order to prevent the making corrupt use of market to increase the price.

### A package of measures for processing

- It is necessary to plan milk collecting network and eliminating unqualified purchasing/collecting points in order to improve the quality of raw milk before it is transported to processors.
- Consistent assessment standard should be established to reduce controversy between dairy farmers and processors.
- Processing plants should be financially sponsored and encouraged to improve their technology and enhance their quality as well as diversify their products. Furthermore, for the better development of the milk processing industry, local authorities are recommended to have policies to support milk processing subsectors such as package production and processing additive.

In conclusion, Value chain upgrading will not only strengthen Vietnamese local companies but also increase the competitiveness of local products against the imported ones. This improvement will offer Vietnamese people to consume more local products with a reasonable price. For the sustainable development of Vietnam dairy sector with higher added value, local enterprises should focus on and analyze the activities in the value chain to eliminate redundancies and implement missing components.

## Author’s information

Dr. Nguyen Viet Khoi has been working for VNU University of Economics and Business at Hanoi, Vietnam since 2001. In the academic year 2012-2013, Dr. Khoi works as post-doc researcher under Fulbright Program Award at Columbia University, USA. Dr. Khoi also worked as visiting researcher and professor at University of Wisconsin - Eau Claire, USA in 2006 and 2008. This author’s main research areas are International Economics, Global Value Chain and E-Commerce. His latest researches have addressed some key issues in MNCs and Trade, Supply Chains under Globalization.

Website: http://ueb.vnu.edu.vn/enNewdetail/44/staff/6141/Nguyen%20Viet%20Khoi.htm

## Authors’ original submitted files for images

Below are the links to the authors’ original submitted files for images. Authors’ original file for figure 1 Authors’ original file for figure 2 Authors’ original file for figure 3 Authors’ original file for figure 4
