# Supplementary material for: Wicked problems: a value chain approach from Vietnam’s dairy product
Source: Springerplus. 2013 Apr 15;2(1):161. doi: 10.1186/2193-1801-2-161 (PMC3647084; doi:10.1186/2193-1801-2-161)
Supplement: Supplementary file 1 — Authors’ original file for figure 1 [file 40064_2013_232_MOESM1_ESM.pdf]

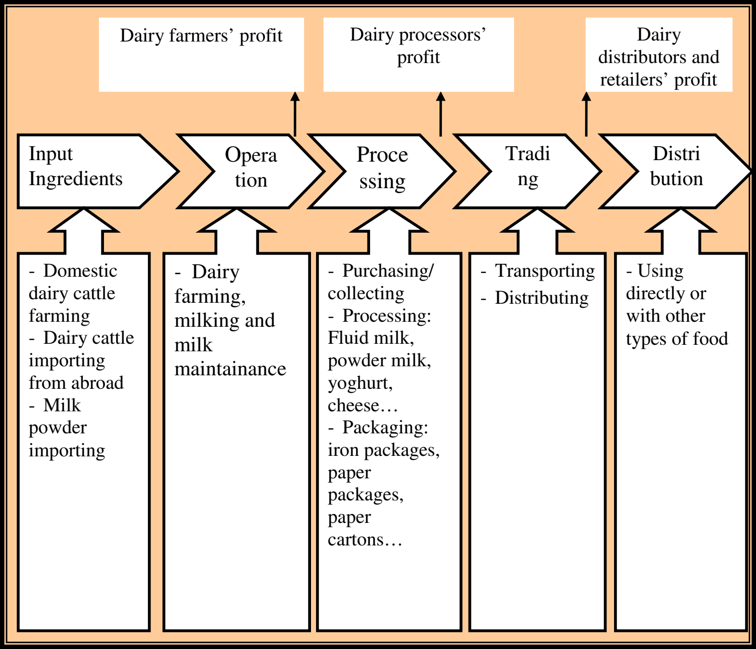
Dairy farmers' profit
Dairy processors' profit
Dairy distributors and retailers' profit
Input Ingredients
Opera tion
Proce ssing
Tradi ng
Distri bution
- Domestic dairy cattle farming
- Dairy cattle importing from abroad
- Milk powder importing
- Dairy farming, milking and milk maintainance
- Purchasing/ collecting
- Processing: Fluid milk, powder milk, yoghurt, cheese…
- Packaging: iron packages, paper packages, paper cartons…
- Transporting
- Distributing
- Using directly or with other types of food
