# Supplementary figures and images for: Wicked problems: a value chain approach from Vietnam’s dairy product
Source: Springerplus. 2013 Apr 15;2(1):161. doi: 10.1186/2193-1801-2-161 (PMC3647084; doi:10.1186/2193-1801-2-161)

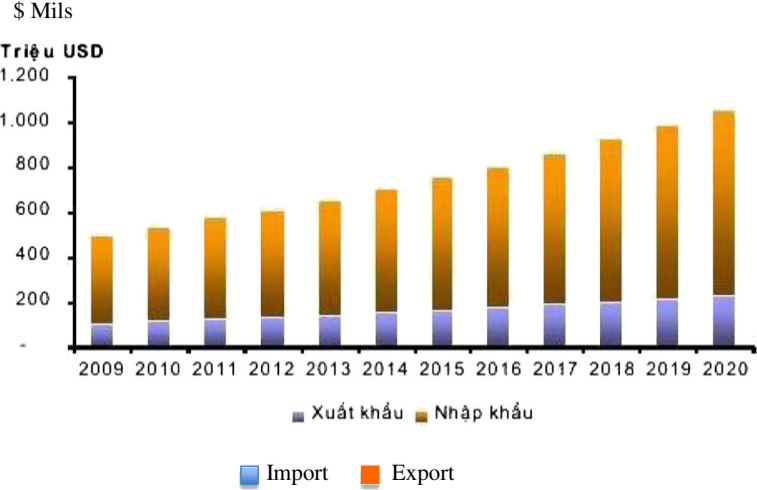

Supplement: Supplementary file 2 — Authors’ original file for figure 2 [file 40064_2013_232_MOESM2_ESM.pdf]

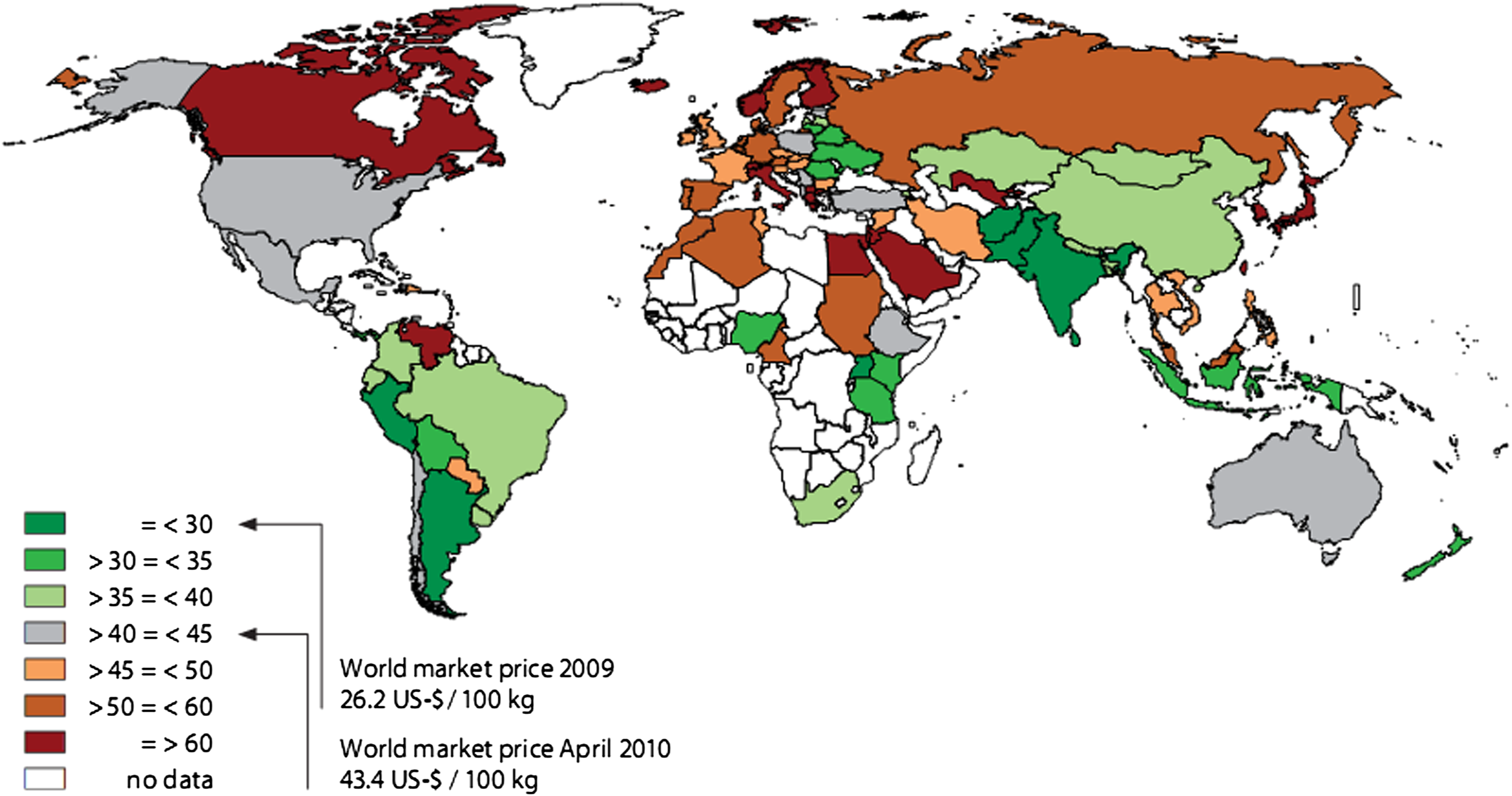

Supplement: Supplementary file 3 — Authors’ original file for figure 3 [file 40064_2013_232_MOESM3_ESM.tiff]

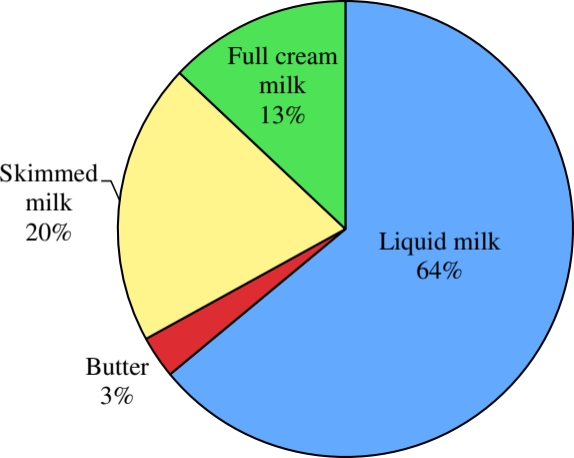

Supplement: Supplementary file 4 — Authors’ original file for figure 4 [file 40064_2013_232_MOESM4_ESM.pdf]
